# Speech and language therapy management of laryngotracheal stenosis – what has changed

**DOI:** 10.1097/MOO.0000000000001111

**Published:** 2026-01-22

**Authors:** Gemma Clunie, Guri Sandhu, Justin Roe

**Affiliations:** a Imperial College London, Faculty of Medicine, Department of Surgery & Cancer; b Imperial College Healthcare NHS Trust, Department of Otolaryngology, Head and Neck Surgery; c The Royal Marsden Hospital NHS Foundation Trust, Department of Speech, Voice and Swallowing, London, UK

**Keywords:** laryngotracheal stenosis, outcomes, speech and language therapy, swallowing, voice

## Abstract

**Purpose of review:**

To examine how the clinical management of adults with laryngotracheal stenosis (LTS) by speech and language therapists (SLTs) has evolved over the past decade. The review highlights changes in assessment, counselling, and rehabilitation practices in response to advances in surgical interventions and explores implications for multidisciplinary care.

**Recent findings:**

Recent literature and clinical experience demonstrate that SLTs play a critical role in managing voice and swallowing outcomes for patients with LTS. While established care pathways exist for reconstructive surgery, the increasing use of minimally invasive endoscopic procedures and complex cases require more nuanced, individualized approaches. Prospective studies have defined the impact of LTS and its treatments on voice and swallowing, and a core outcome set (COS-LTS) has been developed to standardize outcome reporting in future research. Psychosocial support and patient-centred decision-making have become integral components of care.

**Summary:**

Adults with LTS experience multifaceted challenges affecting breathing, voice, and swallowing. SLTs are essential members of the multidisciplinary team, providing pre and postoperative assessment, counselling, and rehabilitation. Advances in treatment options and recognition of psychosocial impacts necessitate flexible, holistic care strategies. Future research should focus on validating outcome measures, implementing the COS-LTS, and incorporating patient priorities to optimize functional and quality-of-life outcomes.

## INTRODUCTION

Adults living with laryngotracheal stenosis (LTS) are affected by a narrowing of their upper airway, anywhere from above the vocal folds to the inferior trachea. Presenting symptoms include dyspnoea, wheeze, stridor and voice changes [1]. The causes of LTS are heterogenous and range from intubation damage, traumatic injuries, radiotherapy, autoimmune disorders, congenital defects and idiopathic disease [2]. Idiopathic subglottic stenosis (iSGS) most commonly occurs in adult women with European ancestry, whose upper airway symptoms are often misdiagnosed as asthma leading to delays in appropriate treatment [3^▪^].

Management of the condition is also heterogenous; with options varying from repeat steroid injections or endoscopic procedures through to complex reconstructive surgeries. The primary aim of treatment is reduction in dyspnoea and decannulation if a tracheostomy is present. However, people with LTS often experience disruption to their swallowing and voice function because of both the underlying condition, and its treatment [4^▪^,^5^]. They also experience significant psychosocial challenges consistent with living with a rare, chronic condition [6^▪▪^]. 

**Box 1:**
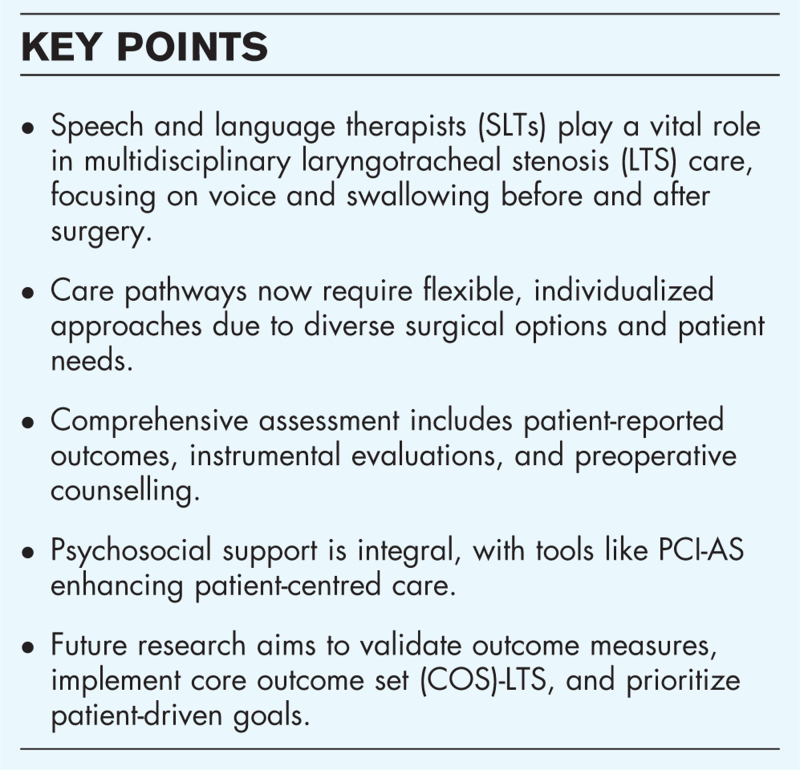
no caption available

Speech and language therapists (SLTs) are core members of the multidisciplinary team (MDT) for adults living with LTS and work closely with expert surgeons and specialist nursing colleagues to support them from diagnosis onwards [7]. The SLT role is to provide assessment of swallowing and voice prior to interventions, counsel people with LTS about the potential impact of any treatment to improve airway patency on their swallowing and voice and then provide ongoing management and rehabilitation for any difficulties that do arise.

**FIGURE 1:**
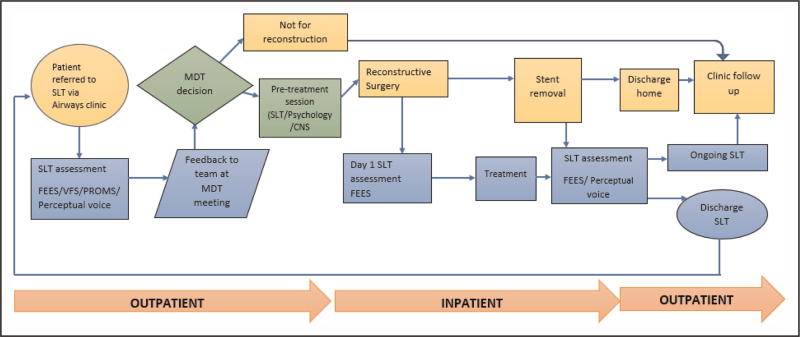
Speech and language therapy care pathway for adults with laryngotracheal stenosis undergoing reconstructive surgery. SLT, speech and language therapy; FEES, flexible endoscopic evaluation of swallowing; VFS, videofluoroscopy; PROMS, patient reported outcome measures; CNS, clinical nurse specialist; MDT, multidisciplinary team (original).

Our centre surgically treats approximately 100 new patients and 800 follow up patients with LTS per year and has had a specialist SLT service since 2016, with a particular clinical and research focus on patients undergoing laryngotracheal reconstruction (LTR) or tracheal resection (TR). This has supported us to understand the lived experience of adults with LTS [8–10] as well as helped to define a robust care pathway (Fig. 1) for swallowing and voice management [4^▪^].

However, as the treatment options for people living with LTS have advanced, many patients can be managed with repeated in-office procedures or endoscopic surgeries, rather than an open reconstructive procedure [11^▪▪^]. Conversely, for patients with intractable stenosis, airway reconstruction surgery can be unsuccessful and require challenging revision procedures [12^▪▪^]. For some, reconstructive procedures are not a viable option due to the concomitant risk to their swallowing or voice. These complexities require SLTs to take a more nuanced approach to care than the care pathway defined in Fig. 1. This paper will explore how the clinical management of adults with LTS has developed for SLTs in the past ten years, alongside key developments in the literature and next steps for research.

## PRE- AND POST-OPERATIVE ASSESSMENT PROTOCOLS

People who undergo head and neck cancer surgery experience a significant postoperative burden, with established pathways in place that include presurgical counselling and assessment as highlighted in national guidelines and standards of care [13,14]. We aim to deliver a similar level of care to our LTS patients. As our service has evolved and based on a series of prospective qualitative and quantitative studies [4^▪^,^9^,^10^] we have better defined our approach to managing people diagnosed with LTS.

Successful surgical management of LTS is underpinned by multidisciplinary, multidimensional assessment of voice and swallowing. LTS itself can lead to altered voice and swallowing, even before surgery [4^▪^]. This is particularly relevant as it informs person-centred decision-making around treatment, especially given the need to navigate the delicate balance between optimizing the airway but with the potential to impact voice quality and swallowing ability.

Medical components of the assessment process for people who are being considered as candidates for complex surgery include: a comprehensive case history with consideration of potential cause of LTS, symptoms, BMI and impact on quality of life (QOL); direct evaluation of the airway under anaesthetic to determine severity and level of stenosis; baseline respiratory function tests and blood tests to rule out underlying vasculitis or inflammatory disease [1]. As highlighted in Fig. 1, the SLT baseline assessment of voice and swallowing includes patient reported outcome measures (PROMS), clinician reported outcome measures and instrumental assessments [flexible endoscopic evaluation of swallowing (FEES), videofluoroscopy, endoscopic evaluation of voice].

## VOICE AND SWALLOWING CONSIDERATIONS

From a voice and swallowing perspective, detailed evaluation can ensure realistic expectations are set regarding outcomes. Preexisting issues can be documented alongside an understanding of likely postoperative trajectories of care. Preoperative guidance will include voice care pre and postsurgery, alongside any relevant exercises to maintain function. For swallowing, even when dysphagia is present it is unusual for patients to require dietary modification prior to surgery due to compensatory strategies but in cases where this is indicated it is recommended [4^▪^].

In the case of reconstructive procedures, patients need to be prepared for a 2-week period of altered communication given the presence of a closed stent and the impact of that procedure on voice quality immediately postsurgery and in the recovery phase. For patients undergoing serial procedures, voice difficulties often follow a pattern of relapse related to their surgeries, likely related to altered airflow and oedema. Voice problems are more likely to persist following reconstructive surgical interventions, with endoscopic procedures having less morbidity [15^▪▪^].

Swallowing is re-assessed at day one postsurgery with FEES. This is to evaluate swallow safety and efficiency. Optimizing analgesia is essential at this stage to ensure pain does not interfere with a potential return to baseline function. While the majority of patients will return to some degree of oral intake at this point, a nasogastric tube is placed during surgery in case additional time is needed for people to transition back to oral intake or if the procedure was particularly complex, for example in the case of the person requiring a pulmonary or hyoid release to facilitate a successful TR and anastomosis.

## PRE-OPERATIVE COUNSELLING

Based on the medical team and SLT assessments, MDT discussion will inform both candidacy and the extent of surgery that may be possible to improve breathing without causing significant compromise to other functional outcomes. Following MDT discussion patients can then be appropriately counselled for the selected surgical option, alongside any potential changes in voice and swallowing function associated with this decision. This augments the shared decision-making process between the patient and clinicians.

Preoperatively, patients who are recommended for a reconstructive surgical procedure, whether an open laryngotracheal reconstruction (LTR), tracheal resection (TR) or Maddern procedure (endoscopic reconstruction) [16^▪^] will meet with SLT, clinical nurse specialists (CNS) and a psychologist. This meeting provides an opportunity to counsel the patient around pre and postoperative expectations to mitigate the psychosocial impacts of the surgery and its consequences, for example short-term alternative feeding, presence of tracheostomy, mucus management [8] and voicelessness. The multidisciplinary nature of this conversation is also vital, to ensure that patients are given a consistent message from all members of the care team, something that has been highlighted in the head and neck literature [17].

## CONSIDERATIONS RELATED TO SPECIFIC AETIOLOGY OF LARYNGOTRACHEAL STENOSIS

Baseline assessment is also essential in an emerging cohort of people diagnosed with late radiation associated LTS [18^▪^]. This is a little-reported condition in the literature but can occur at any, or multiple sites throughout the upper aerodigestive tract. Our experience is that LTS in this cohort is almost always accompanied with late-radiation associated dysphagia (late-RAD) [19]. This therefore requires careful management as optimizing the airway may well impact already impaired swallowing.

Rather than a single procedure, a stepwise approach to surgical intervention is used, for example in the case of step-serial arytenoidectomy for glottic stenosis [20]. Instrumental assessment of swallowing, which can include Flexible Endoscopic Evaluation of Swallowing (FEES) or videofluoroscopy, informs prehabilitation planning to optimize patients for surgery and minimize postoperative complications. It is important to note that swallowing dysfunction does not preclude any surgical intervention for LTS in this patient population, but that the baseline swallowing assessment is vital to maximize the success of the surgery [21].

If there is evidence of ANCA-associated vasculitis (AAV) such as granulomatosis with polyangiitis (GPA), relapsing polychondritis (RP), sarcoidosis, or mucous membrane pemphigoid (MMP) these need to be systemically treated. Treatment comprises immunosuppressants, starting with steroids and often escalating to Rituximab, before complex airway surgery can be considered, always in agreement with specialist medical teams. In some cases, controlling the inflammatory process may lead to longer intervals between airway relapse and fewer endoscopic procedures, eliminating or postponing the need for complex reconstruction [22^▪▪^,23^▪^]. However, SLT involvement requires repeated assessment of function due to the relapsing-remitting profile of their symptoms, with tailored advice to support their voice and swallowing difficulties, alongside psychological support due to the chronic nature of their condition.

At our quaternary airway centre, LTS referrals also include people presenting with cases of traumatic injury, for example ligature (hanging) injuries, or rare but significant laryngeal damage, for example lightning electrocution injuries where the larynx is the exit point. In these cases, thorough, detailed and MDT assessment to provide a clear understanding of existing voice, swallow and airway function is vital. This needs to be balanced alongside the potential changes that may be brought about by surgical intervention to counsel patients effectively, particularly in cases where the injury is unique and the surgical approach similarly atypical [24^▪^].

## ONGOING MANAGEMENT

### Postsurgery

Once oral intake has commenced, regular review by SLT will take place to the point of discharge. In the case of those who have undergone LTR, the stent is removed in theatre at 2 weeks and a further FEES is undertaken the following day, primarily to ensure swallowing safety is optimal and that strategies are in place as appropriate.

Stent removal provides the opportunity to support a return to voicing and the implementation of strategies to avoid hyperfunction. Ideally a skilled voice clinician will be present during the FEES assessment following stent removal, to use the opportunity for an endoscopic evaluation of voice and provide personalized voice therapy recommendations.

### Postdischarge

Given the nature of both LTS and concomitant surgeries, persisting voice and swallowing issues are prevalent, and patients need to be able to access ongoing rehabilitative support as outpatients. From a voice perspective, there may be a requirement to support rehabilitation interventions through phonosurgery, including vocal fold augmentation – however this requires careful management given the fine balance between achieving voice in the context of LTS and reduced airway calibre. Another key component of voice therapy is managing the psychological impacts of an altered voice and breathing patterns – this requires clinicians with requisite experience and skill to work alongside patients to support them with these issues [9,25^▪^].

In those who develop chronic dysphagia, proactive referral to respiratory care can ensure that appropriate assessment has taken place. In addition to sputum analysis and CT scanning in the acute phase, longer term surveillance CT scanning may be recommended to monitor for chronic-progressive lung damage. As part of comprehensive dysphagia management, preventive measures including respiratory physiotherapy, and prophylactic antibiotics may be considered, although patient's need to be carefully selected for this approach [26].

LTS is a lifelong condition for many patients, and voice and swallowing function fluctuate. Repeated endoscopic procedures to optimize airway will always carry a risk to both voice and swallowing, therefore SLT involvement in the care of these patients is advisable. This will include specialist voice assessment and therapy and repeated instrumental swallowing evaluation to inform ongoing management and to facilitate decision making regarding any known dysphonia and dysphagia.

## FUTURE RESEARCH DIRECTIONS

**FIGURE 2:**
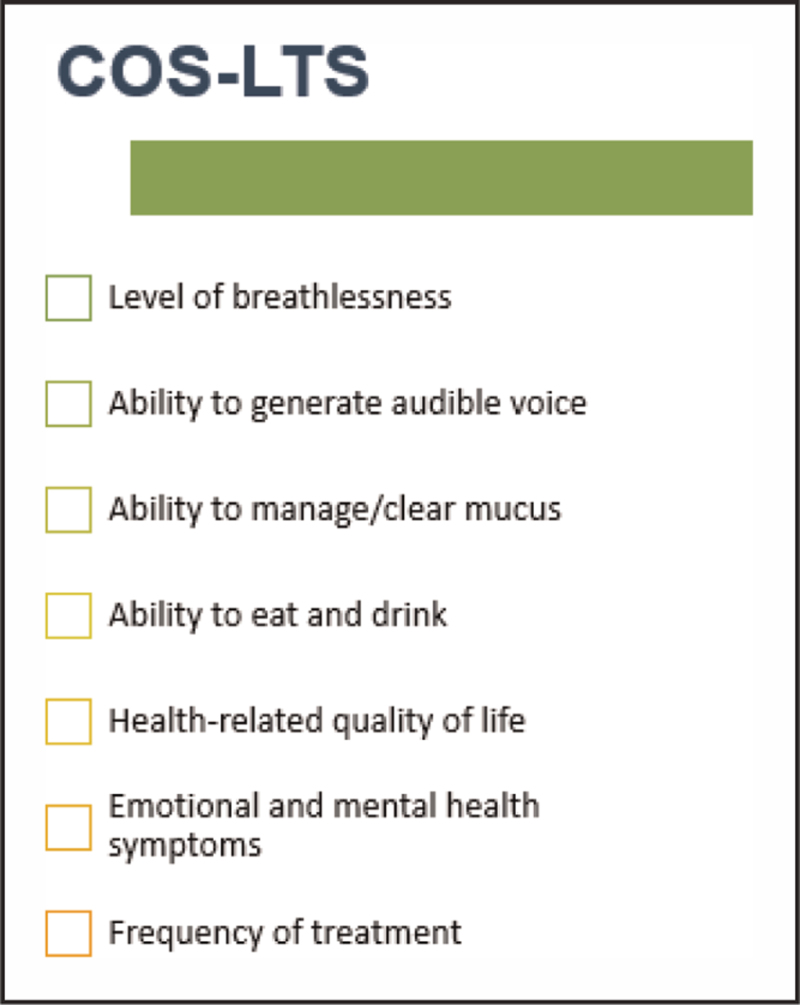
Core outcome set for laryngotracheal stenosis.

Clinical decision making in LTS for SLTs is often challenging due to the heterogeneity of outcome selection in research studies, and the absence of validated outcome measures across a range of different functional domains beyond breathing. A recent Delphi study involving clinicians, researchers and people living with LTS defined a core outcome set (COS) for the condition with seven core outcomes (Fig. 2) identified for use in future research studies [27^▪^].

The COS-LTS is of importance to SLTs because it demonstrates the breadth of domains that need to be included in a clinical evaluation for patients with LTS, and the potential range of functional impairments they may experience, alongside breathing difficulties. The next steps for this work are ongoing and relate to determining which outcome measures are currently used to assess the seven core outcomes, and whether they are sensitive and specific enough for LTS. The identification of validated outcomes for LTS is crucial to future studies to explore the best therapeutic interventions for swallowing and voice difficulties in this population.

Understanding and managing the functional impairments and level of psychosocial distress because of these difficulties is a key component of SLT management of adults living with LTS. Patient concerns inventories (PCIs) have been used in routine outpatient clinics with head and neck cancer patients to allow patients to take more control within their consultations, with a subsequent benefit to QOL [28]. A PCI for people diagnosed with airway stenosis (the PCI-AS) has been developed at our centre [29] and would potentially offer a similar benefit to this patient group, with future clinical testing another key direction for future research. This would support holistic, individualized assessment no matter the underlying cause of the LTS, or the treatment path chosen and hopefully lead to better outcomes for breathing, swallowing, voice and any other difficulties people are experiencing.

A final core component of future research relating to the SLT management of adults with LTS is to continue to engage with patients living with the condition to understand their priorities for forthcoming studies. Living with a chronic, rare disease is challenging and the patient voice is key to both SLT researchers and clinicians identifying the most relevant topics for potential new investigation.

## CONCLUSION

Adults living with LTS experience a range of debilitating and variable symptoms that do not always resolve with surgical, endoscopic or medical treatments. Breathing, swallowing and voice are often affected by both the diagnosis and the treatment of LTS with the complex interplay of these functions a challenging balance to maintain.

SLTs are a key member of the specialist MDT to help support the assessment, management and rehabilitation of these symptoms, in particular swallowing and voice. The care pathway for patients undergoing reconstructive procedures is well defined and has been implemented with success, but those patients with refractory LTS require holistic, individualized care to mitigate the psychosocial burden of living with a chronic condition. SLTs are well placed to advocate for, and work in partnership with their patients to achieve the best outcomes even in these challenging circumstances.

## Acknowledgements

*All authors would like to acknowledge the infrastructure support of the Imperial NIHR Biomedical Research Centre.*

### Financial support and sponsorship

*Gemma Clunie, Senior Clinical Practitioner Research Award Fellow, NIHR304447 is funded by the NIHR for this research project. The views expressed in this publication are those of the author(s) and not necessarily those of the NIHR, NHS or the UK Department of Health and Social Care.*

### Conflicts of interest

*There are no conflicts of interest.*

## REFERENCES AND RECOMMENDED READING

Papers of particular interest, published within the annual period of review, have been highlighted as:

▪ of special interest
▪▪ of outstanding interest
